# Dynamic Changes, Cut-Off Points, Sensitivity, and Specificity of Laboratory Data to Differentiate Macrophage Activation Syndrome from Active Disease

**DOI:** 10.1155/2015/424381

**Published:** 2015-04-30

**Authors:** Raheleh Assari, Vahid Ziaee, Arash Mirmohammadsadeghi, Mohammad-Hassan Moradinejad

**Affiliations:** ^1^Children's Medical Center, Pediatrics Center of Excellence, Tehran, Iran; ^2^Pediatric Rheumatology Research Group, Rheumatology Research Center, Tehran University of Medical Sciences, Tehran, Iran; ^3^Department of Pediatrics, Tehran University of Medical Sciences, Tehran, Iran; ^4^Farabi Eye Research Center, Tehran University of Medical Sciences, Tehran, Iran

## Abstract

*Purpose*. To compare the laboratory data and changes in these data between patients with MAS and patients with flare-up of the autoimmune diseases. *Methods*. In a prospective study, the static laboratory data and dynamic changes in the selected data in 17 consecutive patients with MAS and 53 patients with active disease of SJIA, PJIA, Kawasaki disease, and SLE were compared. The ROC curve analysis was used to evaluate cut-off points, sensitivity, and specificity of the static and dynamic laboratory data to differentiate between MAS and active disease. *Results*. In the MAS group, the mean CRP3, ALT, AST, total bilirubin, ferritin, LDH, PT, PTT, and INR were significantly higher and the mean WBC2, PMN2, Lymph2, Hgb1, 2, 3, ESR2, serum albumin, and sodium were significantly lower than in control group. Some of the important cut-off points were PLT2 < 209000/microliter, AST > 38.5, ALT > 38, WBC < 8200 × 103/UL, ferritin > 5277 ng/mL. *Conclusion*. The dynamic changes in some laboratory data, especially PLT, can differentiate between MAS and active disease. The changes in WBC, PMN, and ESR and the levels of the liver enzymes may also be helpful in the early differentiation. Very high levels of ferritin may also help the diagnosis along with other clinical and laboratory signs.

## 1. Introduction

Macrophage Activation Syndrome (MAS) is a severe, life-threatening disorder. This disease was first described in association with systemic onset juvenile idiopathic arthritis (SJIA) [1] and then described in association with systemic lupus erythematosus (SLE) [2], Kawasaki [3, 4], and other autoimmune disorders. In the recent years, the association between MAS and periodic fever syndromes was also proposed in the literature [5, 6]. The incidence of MAS in rheumatic disorders was about 4.2% and the mortality was 40% [7].

The clinical and laboratory features of the MAS include high fever, hepatic dysfunction, encephalopathy, pancytopenia, bleeding disorders, and high ferritin [8]. The pathognomic feature of the MAS is macrophages in the bone marrow or liver biopsies, actively phagocytizing hematopoietic cells [9].

Because of the similarities between MAS and hemophagocytic lymphohistiocytosis (HLH), some authors believed that the MAS was a secondary or acquired form of HLH [10, 11]. Like HLH, the patients with MAS and juvenile idiopathic arthritis (JIA) had genetic defects in the cytolytic pathway [12, 13]. Also, late onset primary HLH in adult has the specific genetic features [14]. Due to these similarities, the HLH criteria were previously used in the diagnosis of MAS [15]. These criteria were only useful in the end-stage of the MAS, when the treatment was difficult [16, 17]. So, the criteria for the diagnosis of MAS were proposed [8].

Due to progressive and life-threatening course of the MAS, early diagnosis and treatment can be important in reducing morbidity and mortality. The criteria for the MAS diagnosis have some limitations in the early diagnosis. These criteria involves clinical and laboratory findings. The clinical criteria may appear in the later stages of disease [16, 17]. So, these criteria may not be sufficient for early diagnosis. In addition, the laboratory criteria contain only absolute values of data. Because of the inflammatory process in autoimmune diseases, the changes in these laboratory data may be more valuable than absolute values in the diagnosis [16–18]. On the other hand, the need for early differentiation of the MAS attack from the flare-up of the underlying autoimmune diseases made the diagnosis more difficult. Early diagnosis of MAS can reduce the morbidity and mortality.

The aims of the present study were comparing the laboratory data and changes in these data between the patients with MAS and the patients with flare-up of the autoimmune diseases and determine the cut-off points, sensitivity, and specificity of the static and dynamic laboratory data for the early diagnosis of MAS.

## 2. Methods

In a prospective study, 17 consecutive patients diagnosed with MAS, requiring hospital admission, between 2005 and 2014 that were admitted to the Children Medical Center entered the study. The patients over 18 years of age and the patients that did not return for follow-up were excluded from the study. Documented infection was not an exclusion criterion because of the triggering effect of the infection for MAS in the patients with rheumatologic diseases [9]. The study was approved by Institutional Review Board of the Children Medical Center (Tehran University of Medical Sciences). The study and data collection were compliant with the principles of the Declaration of Helsinki. Informed consent was obtained from all patients.

The criteria for diagnosis of the MAS in the patients with SJIA, polyarticular juvenile idiopathic arthritis (PJIA), and Kawasaki disease were obtained from the study of Ravelli and colleagues on the patients with SJIA [8]. The criteria for diagnosis of the MAS in the patients with SLE were obtained from the study of Parodi and colleagues on the patients with SLE [19]. The diagnosis of MAS was based on the clinical criteria, laboratory criteria, and bone marrow aspiration findings. The diagnoses of the SJIA [20], PJIA [20], Kawasaki disease [21], and SLE [22] were based on the relevant criteria.

The control group included 53 patients with the active disease of SJIA, PJIA, Kawasaki disease, and SLE that required hospital admission at the initial diagnosis (before starting treatment) or at the first flare-up. The control group patients were matched with the cases in age and sex. The criteria for active disease in JIA were based on the American College of Rheumatology (ACR) criteria in 2011 [23]. The JIA patients fulfilling moderate or high risk criteria were included in the control group. The criteria for active disease in SLE were based on the study of Bandeira and colleagues [24]. All the patients with Kawasaki disease required hospital admission at the initial diagnosis and did not need active disease criteria.

Age and gender of the patients were noted in the history taking. Bone marrow aspiration was done for all the patients in the MAS group and some of the patients in the control group to confirm the diagnosis.

The previous laboratory records were searched for white blood cell count (WBC), polymorphonuclear (PMN) and lymphocyte (Lymph) absolute count, hemoglobin (Hgb), platelet count (PLT), erythrocyte sedimentation rate (ESR), and C-reactive protein (CRP) in the initial phases of disease. These laboratory data were named as WBC1, PMN1, and so forth. The same laboratory data were obtained from the patients in the onset of MAS (MAS group) or active rheumatologic disease (control group) and named as WBC2, PMN2, and so forth. The same laboratory data were also obtained from the patients 1 month after discharge from the hospital and named as WBC3, PMN3, and so forth. The dynamic changes in these variables were assessed by subtracting the second set of laboratory data from the first and the third sets (e.g., WBC1-WBC2 and WBC3-WBC2). In the patients without previous records, only the second and third groups of data were used in the analysis.

In both groups, the following laboratory data were obtained at the time of first attack of MAS (MAS group) or first admission (control group): serum sodium, blood urea nitrogen (BUN), creatinine, lactate dehydrogenase (LDH), prothrombin time (PT), partial thromboplastin time (PTT), international normalized ratio (INR), total and direct bilirubin, alanine transaminase (ALT), aspartate aminotransferase (AST), alkaline phosphatase (ALP), and ferritin.

All the patients were treated according to the standard treatments for the underlying disease. The patients with MAS were also treated with methylprednisolone pulse (30 mg/kg/day, maximum 1 gram) for 3 days. The resistant or recurrent MAS were treated according to the guidelines of HLH in 2004 with dexamethasone, cyclosporine, and etoposide [15]. The details of the treatment and patients' outcome were not discussed in this paper.

The number of the MAS attacks in the patients was also recorded. In the patients with more than one MAS attack, the data in the first attack were used in the analysis. The static laboratory data and dynamic changes in the selected data were compared between the MAS and control groups.

### 2.1. Statistical Analysis

The statistical analysis was performed with SPSS version 20 (SPSS Inc., Chicago, IL). The Mann-Whitney* U* test was used to assess the difference in quantitative variables between two groups. The receiver operating characteristic (ROC) curve analysis was used to evaluate cut-off points, sensitivity, and specificity of the static and dynamic laboratory data to differentiate between MAS and active disease. ROC curve is a plot with sensitivity on *y*-axis against 1-specificity on *x*-axis. Area under the curve (AUC) is a measure of the overall diagnostic accuracy of tests. The closer the AUC is to 1, the higher is the sensitivity of the test to identify when the disease is truly present. A test with AUC > 0.5 is better than pure chance for differentiating subjects with and without disease [25]. The cut-off point was assigned as the nearest point of the ROC curve to the upper left corner. The level of significance was considered 0.05.

## 3. Results

Seventy patients (17 cases in the MAS group) were included in the study. The characteristics of these patients were summarized in Table 1. In the MAS group, the MAS attack was the first presentation of disease in 9 cases (7 SJIA, 1 SLE, and 1 Kawasaki). In the other 8 cases (4 PJIA, 3 SJIA, and 1 Kawasaki), the MAS attack occurred in the context of the underlying disease. The infection was the triggering factor of the MAS attack in 2 patients. In 4 patients, recurrent attacks of the MAS occurred in the course of follow-up. These recurrences occurred 1–3 years after the first attack.

In the control group, the admission of 31 patients was for the initial diagnosis and treatment. In the other 22 patients, the admission was due to flare-up of the underlying disease.

The mean, standard deviation, and range of the static laboratory data in the case and control groups and *p* value of comparing these data between case and control groups were demonstrated in Table 2. As shown in this table, in the MAS group, the mean CRP3, ALT, AST, total bilirubin, ferritin, LDH, PT, PTT, and INR were significantly higher and the mean WBC2, PMN2, Lymph2, Hgb1, 2, 3, ESR2, serum albumin, and sodium were significantly lower than control group.

The mean, standard deviation, and range of the dynamic changes in the laboratory data (e.g., WBC2-WBC3) in the case and control groups and *p* value of comparing these data between case and control groups were demonstrated in Table 3. As shown in this table, the mean values of the following variables were significantly higher in the MAS group: WBC2-WBC1, WBC3-WBC2, PMN1-PMN2, PMN3-PMN2, Hgb1-Hgb2, PLT1-PLT2, PLT3-PLT2, ESR1-ESR2, and CRP1-CRP2. The mean of the variable “ESR3-ESR2” was significantly lower in the MAS group.

The results of the ROC curve analysis in static and dynamic variables were demonstrated in Tables 4 and 5. The best cut-off point and related sensitivity and specificity of the significant laboratory data were also shown in these tables. The AUC showed significant values for these static variables: WBC2, PMN2, Lymph2, Hb1, 2, PLT1, 2, ESR2, CRP1, 2, ALT, AST, total bilirubin, LDH, ferritin, PT, PTT, INR, serum albumin, and sodium. The AUC showed significant values for these dynamic variables: WBC1-WBC2, WBC3-WBC2, PMN1-PMN2, PMN3-PMN2, Hgb1-Hgb2, PLT1-PLT2, PLT3-PLT2, ESR1-ESR2, ESR3-ESR2, and CRP1-CRP2.

## 4. Discussion

The differentiation between MAS and active diseases is one of the challenging problems in rheumatologic diseases, especially SJIA. These two conditions have overlapping clinical and laboratory features. So, the differentiation can be difficult, especially in early stages.

In this study, the static and dynamic features of the laboratory data were compared in two groups and according to the ROC curve analysis, best cut-off points, sensitivity, and specificity of each variable were determined. It was reported that, in active diseases, when WBC, PLT, and fibrinogen decreased from a high level to normal level, the diagnosis of MAS should be considered [16, 18]. So, the dynamic course of the laboratory data might be more important than the static thresholds for the differentiation between MAS and active disease. In the Delphi survey results, derived from the responses of 352 pediatric rheumatologists to questionnaires, the decreases in the WBC and PLT were among the five most important features in the diagnosis of MAS. But this survey did not contain comparing MAS with active disease, the thresholds of the laboratory data, and their sensitivity and specificity [16]. In Lehmberg and colleagues' study, the decreases in the WBC and PLT were associated with MAS, but their study was without control group and cut-off points [18]. In the recent Kostik and colleagues' study, in the patients with SIJA and MAS, the cut-off points for laboratory data were determined, but the study was retrospective and did not evaluate dynamic course of the laboratory data [26]. Our study was prospective, had control group, evaluated MAS in SJIA, PJIA, SLE, and Kawasaki, and assessed cut-off points for the static and especially dynamic laboratory data. Thus, our study seems unique among the previous studies.

In this study, the highest value of AUC in the ROC curve analysis (Table 4) belonged to the PLT in the time of attack (PLT2). The calculated cut-off point for the PLT2 (206000/microliter) was less than its amount in MAS criteria (226000/microliter) [8]. The static (PLT1 and PLT2) and dynamic (PLT1-PLT2 and PLT3-PLT2) variables about the PLT were significantly different between case and control groups. These differences reflected considerable decrease in the PLT in the MAS attack, comparing active disease. In the MAS diagnosis criteria in SLE, the decrease in PLT was more valuable than the decrease in WBC and Hgb [19]. Thus, the decrease in the PLT might be the best laboratory data in the early diagnosis of MAS and its differentiation from active disease. At the time of attack, the PLT < 206000/microliter and the decrease in PLT > 30000/microliter (from the previous records) could differentiate between MAS and active disease.

After PLT2, the most value of AUC belonged to the liver function tests (AST and ALT). The cut-off point for AST was 38.5 U/L and for ALT was 38 U/L. AST > 59 was a part of the MAS criteria [8]. In the Delphi survey, the increase in the liver enzymes was also among the nine important criteria in the diagnosis of MAS [16]. These studies showed that the AST and ALT might be important in the early diagnosis of MAS. Further studies should be done to evaluate value of dynamic changes in the liver function test for diagnosis of MAS.

The value of the AUC for albumin was as high as the value for AST and ALT. The calculated cut-off point for albumin in our study (3 mg/dL) was near the cut-off point in Ravelli and colleagues' study (2.5 mg/dL) [8]. Albumin is a negative acute phase protein. In contrast with the other acute phase proteins, the serum level of the albumin decreased in response to the inflammatory cytokines [27]. So, the decrease in albumin might alarm the beginning of a severe inflammatory phase, like MAS. The AUC for the other serum protein, fibrinogen, was not significant. The level of fibrinogen decreased only in end-stages of the MAS, when consumption of coagulopathy and disseminated intravascular coagulation occurred [28]. So, the fibrinogen level was not valuable for early diagnosis of the MAS in our study.

The AUC values were also significant for WBC2 and PMN2. In addition, these variables and their related dynamic variables (WBC2-WBC1, WBC3-WBC2, PMN1-PMN2, and PMN3-PMN2) were significantly different between case and control groups. The cut-off points for these variables (8200 × 10^3^/UL for WBC2 and 3900 × 10^3^/UL for PMN2) were higher than the cut-off points in other studies. These higher values might reflect that, in our patients, the diagnosis of MAS was done in an early stage with the changes in general condition, decrease in PLT, increase in liver enzymes and ferritin, and presence of hemophagocytes in the bone marrow. In this early stage, the WBC and PMN values were still in higher levels than later stages of disease. So, the higher cut-off points for WBC and PMN could be helpful for early diagnosis of MAS. In addition, the decrease in WBC > 3570 × 10^3^/UL and the decrease in PMN > 1938 × 10^3^/UL at the time of attack could also differentiate between MAS and active disease.

The lymphocyte count at the time of attack (Lymph2) had also high AUC value. This variable was also significantly lower in MAS group than control group. But the dynamic changes in this variable did not show significant difference between two groups. So, only lymphocyte counts at the time of MAS attack could help the diagnosis and the dynamic changes might not be helpful.

All three static Hgb variables and the dynamic variable Hgb1-Hgb2 were significantly different between two groups. The cut-off point for Hgb2 (8.9 g/dL) was similar to the cut-off point in the study of Kostik and colleagues (9 g/dL). So, the Hgb < 8.9 g/dL at the time of attack or decrease in Hgb > 0.55 g/dL from the previous records could help the diagnosis of MAS.

In contrast with other studies, the AUC for ferritin showed also high values. The cut-off point for ferritin was 5277 ng/mL. In the HLH criteria, the minimum threshold of ferritin for the diagnosis of HLH was 500 ng/mL [15]. This value was also considered as a MAS diagnosis criteria in SLE [19]. Ferritin is a proinflammatory mediator that causes cytokine storm [29]. The levels of ferritin are high in both MAS and active disease, even higher than 5000 ng/mL. So, the threshold of 500 ng/mL might not differentiate between MAS and active disease and higher thresholds (>5277 ng/mL), if present, might help the diagnosis along with other signs and laboratory data.

In this study, the AUC for CRP2 was not significant. CRP1 and CRP2 showed no significant differences between case and control groups. In contrast, in previous studies, CRP had higher levels in MAS than in HLH [18]. In our study, only one dynamic variable (CRP1-CRP2) was significantly higher in MAS group. So, the absolute levels of CRP might not be helpful in diagnosis of MAS and only dynamic changes might differentiate between MAS and active disease. On the other hand, ESR at the time of attack (ESR2) and its dynamic variable (ESR1-ESR2 and ESR3-ESR2) showed high AUC and significant difference between case and control groups. So, the decrease in ESR might be a better variable than increase in CRP for diagnosis of the MAS.

The CRP value at the remission (CRP3) was also significantly higher in MAS group. The previous studies did not consider the laboratory data in the remission phase. CRP is a positive acute phase protein that is made in response to the cytokines released by macrophages [30]. In the MAS, a cytokine storm occurred. So, in the remission phase of MAS, CRP might decrease with a slower rate than active disease and might be higher in the remission phase. Thus, high CRP in the remission phase might be a sign of previous MAS attack.

The AUC for serum sodium showed high levels, with a cut-off point of 133 meq/L. This cut-off point was 130 meq/L in the Ravelli and colleagues' study [8] and 137 meq/L in the Kostik and colleagues' study [26]. Lower serum sodium was considered a sign for the diagnosis of MAS in the Ravelli and colleagues' study [8]. Lower serum sodium in MAS attack might be caused by syndrome of inappropriate antidiuretic hormone secretion (SIADH) [31] or cytokine effects on the proximal tubule and resultant disturbance in sodium reabsorption [32]. The early decrease of sodium in the acute phase of the MAS may reflect aggravating general condition of the patient. So, lower serum sodium could alert the examiner for the immediate treatment for the MAS.

The main limitation of our study was low sample size. Higher sample size might yield more accurate results. Furthermore, the increase in the sCD163 and the decrease in C3 and C4, gene expression profiling (not evaluated in our study), were considered helpful in the differentiation of MAS from active disease in some studies. These laboratory exams may not be cost-effective and available in all centers. Further studies should be done to evaluate value of these laboratory exams in the diagnosis of MAS.

In conclusion, the dynamic changes in some laboratory data, especially PLT, can differentiate between MAS and active disease in the early phases with high sensitivity and specificity. The changes in WBC, PMN, and ESR and the levels of the liver enzymes may also be helpful in the early differentiation. Very high levels of ferritin may also help the diagnosis along with other clinical and laboratory signs.

## Figures and Tables

**Table 1:** The characteristics of patients. The quantitative variable (age) was shown as mean ± SD (range). The qualitative variables were shown as number (percentage).

	MAS (*n* = 17)	Control (*n* = 53)
Age		
Mean ± SD (range)	7.44 ± 4.4 (1–17)	6.6 ± 3.93 (1–17)
Gender		
Male	10 (58.8%)	38 (71.7%)
Female	7 (41.2%)	15 (28.3%)
Diagnosis		
SJIA	10 (58.8%)	31 (58.3%)
PJIA	4 (23.5%)	12 (22.6%)
Kawasaki	2 (11.8%)	6 (11.3%)
SLE	1 (5.9%)	4 (7.5%)
Presentation		
First presentation	9 (52.9%)	31 (58.5%)
On background illness	8 (47.1%)	22 (41.5%)

MAS: Macrophage Activation Syndrome, SD: standard deviation, SJIA: systemic onset juvenile idiopathic arthritis, PJIA: polyarticular juvenile idiopathic arthritis, and SLE: systemic lupus erythematosus.

**Table 2:** The mean, standard deviation, and range of the laboratory data in the case and control groups and *p* value of comparing these data between case and control groups.

	MAS	Control (active disease)	*p* value
WBC1 (×10^3^/uL)	16122 ± 9105 (6100–38121)	16280 ± 10857 (2520–61300)	1
WBC2 (×10^3^/uL)	8784 ± 10310 (520–38430)	15679 ± 8502 (700–41200)	**0.001**
WBC3 (×10^3^/uL)	15617 ± 12602 (1130–46670)	10299 ± 4551 (2140–25500)	0.2
PMN1 (×10^3^/uL)	11664 ± 8500 (1449–34080)	11174 ± 7870 (1128–36556)	0.4
PMN2 (×10^3^/uL)	6775 ± 6569 (120–18069)	11377 ± 7536 (583–35020)	**0.02**
PMN3 (×10^3^/uL)	11472 ± 9539 (1142–31408)	5834 ± 3462 (1001–17110)	0.07
Lymph1 (×10^3^/uL)	4046 ± 4477 (866–19500)	3815 ± 4441 (1108–32550)	0.9
Lymph2 (×10^3^/uL)	2638 ± 3731 (639–15333)	3635 ± 1812 (1209–8694)	<**0.001**
Lymph3 (×10^3^/uL)	3356 ± 2600 (550–8390)	3501 ± 1917 (107–11272)	0.4
Hgb1 (g/dL)	9.13 ± 1.45 (6.80–12.20)	10.40 ± 1.40 (7.50–14.3)	**0.003**
Hgb2 (g/dL)	7.96 ± 1.66 (6.10–11.4)	10.33 ± 1.66 (7.30–14.5)	<**0.001**
Hgb3 (g/dL)	8.95 ± 1.16 (6.90–10.70)	11.58 ± 1.91 (6.50–15)	<**0.001**
PLT1 (/microliter)	292588 ± 170526 (29000–638000)	489365 ± 212249 (138000–1144000)	**0.001**
PLT2 (/microliter)	105882 ± 91791 (9000–406000)	502617 ± 189080 (56000–1010000)	<**0.001**
PLT3 (/microliter)	321785 ± 177361 (44000–770000)	393512 ± 143221 (68000–779000)	0.07
ESR1 (mm/h)	69.53 ± 40.96 (9–126)	73.67 ± 30.24 (5–125)	0.7
ESR2 (mm/h)	46.81 ± 49.47 (2–165)	72.21 ± 35.82 (6–148)	**0.008**
ESR3 (mm/h)	37.53 ± 30.176 (10–102)	23.78 ± 27.85 (3–140)	0.1
CRP1 (mg/L)	37 ± 23.61 (5–75)	68 ± 52 (0–246)	0.3
CRP2 (mg/L)	139 ± 101 (40–345)	96 ± 70 (22–317)	0.1
CRP3 (mg/L)	32.84 ± 46 (0.5–168)	14.91 ± 19.4 (0–75)	**0.03**
ALT (u/L)	315 ± 840 (12–3560)	28.28 ± 31.4 (6–187)	<**0.001**
AST (u/L)	583 ± 1506 (17–6300)	32.2 ± 21.7 (10–142)	<**0.001**
ALP (u/L)	409 ± 290 (165–1220)	392 ± 171 (84–941)	0.3
ALB (gr/dL)	3.03 ± 0.69 (2–4.3)	3.80 ± 0.62 (2.7–4.7)	**0.002**
Total Pr (gr/dL)	6.42 ± 1.77 (4–8.7)	6.90 ± 1.04 (4.9–8.4)	0.5
BilT (mg/dL)	5.36 ± 7.72 (0.4–24.3)	0.88 ± 0.91 (0.4–2.7)	**0.050**
BilD (mg/dL)	3.32 ± 4.87 (0.1–14)	0.26 ± 0.16 (0.2–0.6)	0.2
Ferritin (ng/mL)	60590 ± 73343 (1617–200000)	5253 ± 5505 (300–18035)	**0.009**
Fibrinogen (mg/dL)	517 ± 641 (130–4944)	489 ± 94 (423–556)	0.2
LDH (Iu/L)	1913 ± 1516 (136–4944)	679 ± 336 (258–1318)	**0.02**
BUN (mg/dL)	16.75 ± 11.94 (5–41)	10.18 ± 4.03 (4–26)	0.1
Cr (mg/dL)	0.60 ± 0.39 (0.12–1.80)	0.55 ± 0.11 (0.3–0.8)	0.5
Na (meq/L)	130 ± 6.3 (115–137)	136 ± 4.3 (128–144)	**0.004**
TG (mg/dL)	329 ± 188 (109–727)	161 ± 119 (42–280)	0.1
Chol (mg/dL)	224 ± 130 (112–530)	181 ± 131 (88–274)	0.7
PT	18.06 ± 7.7 (13–43)	16.4 ± 7.9 (12–34)	**0.04**
PTT	43 ± 14 (18–75)	31.50 ± 9.44 (13–45)	**0.006**
INR	1.67 ± 0.6 (1–2.7)	1.16 ± 0.26 (1–1.8)	**0.009**

MAS: Macrophage Activation Syndrome, WBC: white blood cells, PMN: polymorphonuclear, Lymph: lymphocytes, Hgb: hemoglobin, PLT: platelet, ESR: erythrocyte sedimentation rate, CRP: C-reactive protein, ALT: alanine aminotransferase, AST: aspartate aminotransferase, ALP: alkaline phosphatase, ALB: albumin, Total Pr: total protein, Bil T: total bilirubin, Bil D: direct bilirubin, LDH: lactate dehydrogenase, BUN: blood urea nitrogen, Cr: creatinine, Na: natrium, TG: triglycerides, Chol: cholesterol, PT: prothrombin time, PTT: partial prothrombin time, and INR: international normalized ratio. The previous laboratory records: WBC1, PMN1, and so forth. The laboratory data at the onset of MAS (MAS group) or active rheumatologic disease (control group): WBC2, PMN2, and so forth. The laboratory data 1 month after discharge from the hospital: WBC3, PMN3, and so forth.

**Table 3:** Differences between parameters detected at 2 different times in the case and control groups and *p* value of comparing data between these two groups. The values are reported as mean ± standard deviation (range).

Parameter	MAS	Control	*p* value
WBC1-WBC2 (×10^3^/uL)	7337 ± 11572 (−19030 to 34191)	781.04 ± 9243 (−24000 to 43500)	**0.004**
WBC3-WBC2 (×10^3^/uL)	8284 ± 14647 (−19640 to 44070)	−5593 ± 7941 (−28800 to 9830)	**0.00**
PMN1-PMN2 (×10^3^/uL)	4888–10049 (−16228 to 31407)	−137 ± 6127 (−16908 to 12555)	**0.02**
PMN3-PMN2 (×10^3^/uL)	6404 ± 9787 (−3750 to 29622)	−6167 ± 6288 (−21261 to 3572)	<**0.001**
Lymph1-Lymph2 (×10^3^/uL)	1277 ± 5889 (−11763 to 17670)	381.78 ± 4864 (−6972 to 28278)	0.07
Lymph3-Lymph2 (×10^3^/uL)	1484 ± 2505 (−1151 to 6560)	135.45 ± 2504 (−6927 to 8025)	0.1
Hgb1-Hgb2 (g/dL)	1.24 ± 1.34 (−1.10 to 4.20)	0.067 ± 1.38 (−2.50 to 2.90)	**0.008**
Hgb3-Hgb2 (g/dL)	1.17 ± 1.45 (−1.50 to 3.10)	1.17 ± 1.75 (−2.40 to 5.10)	0.8
PLT1-PLT2 (/microliter)	190176 ± 176530 (11000–485000)	−2826 ± 176163 (−377000 to 353000)	**0.001**
PLT3-PLT2 (/microliter)	217785 ± 194694 (−2000 to 75800)	−90864 ± 164212 (−633000 to 140000)	<**0.001**
ESR1-ESR2 (mm/h)	28 ± 35 (−40 to 84)	2.65 ± 35.53 (−70 to 74)	**0.03**
ESR3-ESR2 (mm/h)	3.5 ± 60 (−91 to 155)	49.68 ± 40.11 (−42 to 115)	**0.002**
CRP1-CRP2 (mg/L)	97.11 ± 93.75 (20 to 303)	27.61 ± 83.73 (−174 to 252)	**0.003**
CRP3-CRP2 (mg/L)	116.95 ± 115.22 (0 to 299)	80.20 ± 64.38 (21.60 to 269)	0.5

MAS: Macrophage Activation Syndrome, WBC: white blood cells, PMN: polymorphonuclear, Lymph: lymphocytes, Hgb: hemoglobin, PLT: platelet, ESR: erythrocyte sedimentation rate, and CRP: C-reactive protein. The previous laboratory records: WBC1, PMN1, and so forth. The laboratory data at the onset of MAS (MAS group) or active rheumatologic disease (control group): WBC2, PMN2, and so forth. The laboratory data 1 month after discharge from the hospital: WBC3, PMN3, and so forth.

**Table 4:** The results of receiver operating characteristic (ROC) curve analysis for static variables.

Parameter	ROC-AUC	*p* value	Cut-off value	Sensitivity	Specificity	95% confidence interval
WBC1 (×10^3^/uL)	0.50	0.96	—	—	—	0.33–0.66
WBC2 (×10^3^/uL)	0.77	0.001	≤8.2 × 10^9^	70%	86%	0.61–0.93
PMN1 (×10^3^/uL)	0.08	0.9	—	—	—	0.34–0.67
PMN2 (×10^3^/uL)	0.70	0.02	≤3.9 × 10^9^	60%	85%	0.53–0.86
Lymph1 (×10^3^/uL)	0.50	0.9	—	—	—	0.33–0.68
Lymph2 (×10^3^/uL)	0.83	<0.001	≤1.8 × 10^9^	81%	99.94%	0.67–0.99
Hgb1 (g/dL)	0.733	0.004	≤**9.2**	58%	83%	0.58–0.87
Hgb2 (g/dL)	0.83	<0.001	≤**8.9**	76%	79%	0.70–0.96
PLT1 (/microliter)	0.76	0.001	≤**316500**	58%	79%	0.63–0.89
PLT2 (/microliter)	0.96	<0.001	≤**204000**	94%	99.96%	0.92–1.00
ESR1 (mm/h)	0.41	0.3	—	—	—	0.22–0.60
ESR2 (mm/h)	0.78	0.001	≤**54**	81%	72%	0.65–0.92
CRP1 (mg/L)	0.71	0.03	≤**47.7**	61%	69%	0.54–0.85
CRP2 (mg/L)	0.63	0.1	≥103	58%	69%	0.47–0.79
ALT (u/L)	0.85	<0.001	≥**38**	82%	84%	0.73–0.97
AST (u/L)	0.87	<0.001	≥**38.5**	82%	78%	0.76–0.98
ALP (U/L)	0.42	0.4	—	—	—	—
ALB (gr/dl)	0.86	0.001	≤**3.0**	75%	90%	0.72–1.00
BilT (mg/dL)	0.81	0.04	≥**0.98**	66%	84%	0.59–1.00
BilD (mg/dL)	0.69	0.2	—	—	—	0.42–0.96
Ferritin (ng/mL)	0.81	0.01	≥**5277**	92%	73%	0.63–0.99
Fibrinogen (mg/dL)	0.69	0.2	—	—	—	0.59–1.00
LDH (Iu/L)	0.80	0.02	≥**829**	72%	80%	0.63–1.00
BUN (mg/dL)	0.63	0.1	—	—	—	0.46–0.81
Cr (mg/dL)	0.44	0.6	—	—	—	0.25–0.64
Na (meq/L)	0.81	0.005	≤**133**	70%	75%	0.67–0.95
TG (mg/dL)	0.79	0.1	—	—	—	0.49–1.00
Chol (mg/dL)	0.61	0.6	—	—	—	0.064–1.00
PT	0.74	0.04	≥**14.7**	61.5%	75%	0.53–0.95
PTT	0.79	0.01	≥**39**	61	99	0.62–0.97
INR	0.80	0.01	≥**1.15**	84%	67%	0.62–0.97

ROC-AUC: receiver operating characteristic-area under the curve, MAS: Macrophage Activation Syndrome, WBC: white blood cells, PMN: polymorphonuclear, Lymph: lymphocytes, Hgb: hemoglobin, PLT: platelet, ESR: erythrocyte sedimentation rate, CRP: C-reactive protein, ALT: alanine aminotransferase, AST: aspartate aminotransferase, ALB: albumin, Total Pr: total protein, Bil T: total bilirubin, Bil D: direct bilirubin, LDH: lactate dehydrogenase, BUN: blood urea nitrogen, Cr: creatinine, Na: natrium, TG: triglycerides, Chol: cholesterol, PT: prothrombin time, PTT: partial prothrombin time, and INR: international normalized ratio. The previous laboratory records: WBC1, PMN1, and so forth. The laboratory data at the onset of MAS (MAS group) or active rheumatologic disease (control group): WBC2, PMN2, and so forth. The laboratory data 1 month after discharge from the hospital: WBC3, PMN3, and so forth.

**Table 5:** The results of receiver operating characteristic (ROC) curve analysis for dynamic variables.

	ROC-AUC	*p* value	Cut-off value	Sensitivity	Specificity	95% confidence interval
WBC1-WBC2 (×10^3^/uL)	0.73	0.004	**3570**	70%	77%	0.59–0.88
WBC3-WBC2 (×10^3^/uL)	0.84	<0.001	**195**	78%	77%	0.7–0.98
PMN1-PMN2 (×10^3^/uL)	0.69	0.02	**1938**	73%	70%	0.53–0.86
PMN3-PMN2 (×10^3^/uL)	0.90	<0.001	**−243**	84%	86%	0.81–0.99
Lymph1-Lymph2 (×10^3^/uL)	0.65	0.09	—	—	—	—
Lymph3-Lymph2 (×10^3^/uL)	0.65	0.08	—	—	—	—
Hgb1-Hgb2 (g/dL)	0.72	0.008	**0.55**	76%	68%	0.58–0.85
Hgb3-Hgb2 (g/dL)	0.52	0.8	—	—	—	—
PLT1-PLT2 (/microliter)	0.78	0.001	**30000**	82%	60%	0.67–0.90
PL3-PLT2 (/microliter)	0.92	<0.001	**97000**	78%	90%	0.85–1.00
ESR1-ESR2 (mm/h)	0.69	0.02	**9**	64%	56%	0.54–0.85
ESR3-ESR2 (mm/h)	0.80	0.002	−**4.5**	75%	86%	0.63–0.97
CRP1-CRP2 (mg/L)	0.77	0.003	**42.5**	76%	67%	0.64–0.89
CRP3-CRP2 (mg/L)	0.53	0.5	—	—	—	—

ROC-AUC: receiver operating characteristic-area under the curve, MAS: Macrophage Activation Syndrome, WBC: white blood cells, PMN: polymorphonuclear, Lymph: lymphocytes, Hgb: hemoglobin, PLT: platelet, ESR: erythrocyte sedimentation rate, and CRP: C-reactive protein. The previous laboratory records: WBC1, PMN1, and so forth. The laboratory data at the onset of MAS (MAS group) or active rheumatologic disease (control group): WBC2, PMN2, and so forth. The laboratory data 1 month after discharge from the hospital: WBC3, PMN3, and so forth.

## Abbreviations

MAS: Macrophage Activation Syndrome
WBC: White blood cells
PMN: Polymorphonuclear
Lym: Lymphocytes
Hgb: Hemoglobin
PLT: Platelet
ESR: Erythrocyte sedimentation rate
CRP: C-reactive protein
ALT: Alanine aminotransferase
AST: Aspartate aminotransferase
LDH: Lactate dehydrogenase
PT: Prothrombin time
PTT: Partial prothrombin time
INR: International normalized ratio
ROC: Receiver operating characteristic
WBC1, PMN1, and so forth: The previous laboratory records
WBC2, PMN2, and so forth: The laboratory data at the onset of MAS (MAS group) or active rheumatologic disease (control group)
WBC3, PMN3, and so forth: The laboratory data 1 month after discharge from the hospital.

## References

[1] Prieur A.-M., Stephan J. L. (1994). Macrophage activation syndrome in children with joint disease. *Revue du Rhumatisme—English Edition*.

[2] Parodi A., Davì S., Pringe A. B. (2009). Macrophage activation syndrome in juvenile systemic lupus erythematosus: a multinational multicenter study of thirty-eight patients. *Arthritis & Rheumatism*.

[3] Latino G. A., Manlhiot C., Yeung R. S. M., Chahal N., McCrindle B. W. (2010). Macrophage activation syndrome in the acute phase of Kawasaki disease. *Journal of Pediatric Hematology/Oncology*.

[4] Simonini G., Pagnini I., Innocenti L., Calabri G. B., De Martino M., Cimaz R. (2010). Macrophage activation syndrome/hemophagocytic lymphohistiocytosis and Kawasaki disease. *Pediatric Blood & Cancer*.

[5] Rigante D., Capoluongo E., Bertoni B. (2007). First report of macrophage activation syndrome in hyperimmunoglobulinemia D with periodic fever syndrome. *Arthritis and Rheumatism*.

[6] Rossi-Semerano L., Hermeziu B., Fabre M., Koné-Paut I. (2011). Macrophage activation syndrome revealing familial mediterranean fever. *Arthritis Care and Research*.

[7] Moradinejad M. H., Ziaee V. (2011). The incidence of macrophage activation syndrome in children with rheumatic disorders. *Minerva Pediatrica*.

[8] Ravelli A., Magni-Manzoni S., Pistorio A. (2005). Preliminary diagnostic guidelines for macrophage activation syndrome complicating systemic juvenile idiopathic arthritis. *Journal of Pediatrics*.

[9] Ravelli A., Martini A., Lehman T. H., Cimaz R. (2008). Macrophage activation syndrome. *Pediatric Rheumatology*.

[10] Athreya B. H. (2002). Is macrophage activation syndrome a new entity?. *Clinical and Experimental Rheumatology*.

[11] Ramanan A. V., Schneider R. (2003). Macrophage activation syndrome—what's in a name!. *Journal of Rheumatology*.

[12] Ravelli A., Grom A. A., Behrens E. M., Cron R. Q. (2012). Macrophage activation syndrome as part of systemic juvenile idiopathic arthritis: diagnosis, genetics, pathophysiology and treatment. *Genes and Immunity*.

[13] Canna S. W., de Jesus A. A., Gouni S. (2014). An activating NLRC4 inflammasome mutation causes autoinflammation with recurrent macrophage activation syndrome. *Nature Genetics*.

[14] Wang Y., Wang Z., Zhang J. (2014). Genetic features of late onset primary hemophagocytic lymphohistiocytosis in adolescence or adulthood. *PLoS ONE*.

[15] Henter J.-I., Horne A., Aricó M. (2007). HLH-2004: diagnostic and therapeutic guidelines for hemophagocytic lymphohistiocytosis. *Pediatric Blood and Cancer*.

[16] Davì S., Consolaro A., Guseinova D. (2011). An international consensus survey of diagnostic criteria for macrophage activation syndrome in systemic juvenile idiopathic arthritis. *Journal of Rheumatology*.

[17] Davi S., Lattanzi B., Demirkaya E. (2012). Toward the development of new diagnostic criteria for macrophage activation syndrome in systemic juvenile idiopathic arthritis. *Annals of Paediatric Rheumatology*.

[18] Lehmberg K., Pink I., Eulenburg C., Beutel K., Maul-Pavicic A., Janka G. (2013). Differentiating macrophage activation syndrome in systemic juvenile idiopathic arthritis from other forms of hemophagocytic lymphohistiocytosis. *Journal of Pediatrics*.

[19] Parodi A., Davì S., Pringe A. B. (2009). Macrophage activation syndrome in juvenile systemic lupus erythematosus: a multinational multicenter study of thirty-eight patients. *Arthritis and Rheumatism*.

[20] Petty R. E., Southwood T. R., Manners P. (2004). International League of Associations for Rheumatology classification of juvenile idiopathic arthritis: second revision, Edmonton, 2001. *Journal of Rheumatology*.

[21] Newburger J. W., Takahashi M., Gerber M. A. (2004). Diagnosis, treatment, and long-term management of Kawasaki disease: a statement for health professionals from the Committee on Rheumatic Fever, Endocarditis and Kawasaki Disease, Council on Cardiovascular Disease in the Young, American Heart Association. *Circulation*.

[22] Hochberg M. C. (1997). Updating the American College of Rheumatology revised criteria for the classification of systemic lupus erythematosus. *Arthritis and rheumatism*.

[23] Beukelman T., Patkar N. M., Saag K. G. (2011). 2011 American College of Rheumatology recommendations for the treatment of juvenile idiopathic arthritis: initiation and safety monitoring of therapeutic agents for the treatment of arthritis and systemic features. *Arthritis Care & Research*.

[24] Bandeira M., Buratti S., Bartoli M. (2006). Relationship between damage accrual, disease flares and cumulative drug therapies in juvenile-onset systemic lupus erythematosus. *Lupus*.

[25] Park S. H., Goo J. M., Jo C.-H. (2004). Receiver operating characteristic (ROC) curve: practical review for radiologists. *Korean Journal of Radiology*.

[26] Kostik M. M., Dubko M. F., Masalova V. V. (2015). Identification of the best cutoff points and clinical signs specific for early recognition of macrophage activation syndrome in active systemic juvenile idiopathic arthritis. *Seminars in Arthritis & Rheumatism*.

[27] Ritchie R. F., Palomaki G. E., Neveux L. M., Navolotskaia O., Ledue T. B., Craig W. Y. (1999). Reference distributions for the negative acute-phase serum proteins, albumin, transferrin, and transthyretin: a practical, simple and clinically relevant approach in a large cohort. *Journal of Clinical Laboratory Analysis*.

[28] Benedetti F. D., Schneider R., Cassidy J. T., Petty R. E., Laxer R. M., Lindsley C. B. (2011). Systemic juvenile idiopathic arthritis. *Textbook of Clinical Rheumatology*.

[29] Rosário C., Zandman-Goddard G., Meyron-Holtz E. G., D'Cruz D. P., Shoenfeld Y. (2013). The hyperferritinemic syndrome: macrophage activation syndrome, Still's disease, septic shock and catastrophic antiphospholipid syndrome. *BMC Medicine*.

[30] Pepys M. B., Hirschfield G. M. (2003). C-reactive protein: a critical update. *Journal of Clinical Investigation*.

[31] Kounami S., Yoshiyama M., Nakayama K. (2005). Macrophage activation syndrome in children with systemic-onset juvenile chronic arthritis. *Acta Haematologica*.

[32] Yamazawa K., Kodo K., Maeda J. (2006). Hyponatremia, hypophosphatemia, and hypouricemia in a girl with macrophage activation syndrome. *Pediatrics*.

